# Hyaluronic Acid Fillers Versus Polynucleotides for Under-Eye Rejuvenation

**DOI:** 10.3390/jcm15134971

**Published:** 2026-06-26

**Authors:** Rabia S. Khan, Kashif Hafeez

**Affiliations:** 1 ICE Postgraduate Dental Institute and Hospital, 24 Furness Quay, Salford M50 3XZ, UK; 2 Dental Faculty Oral & Craniofacial Sciences, King’s College London, London SE1 9SP, UK; kashif.hafeez@kl.ac.uk

**Keywords:** periorbital rejuvenation, hyaluronic acid fillers, polynucleotides, polydeoxyribonucleotides (PDRN), tear trough deformity, dermal remodelling, skin quality, regenerative aesthetics

## Abstract

The periorbital region represents one of the most challenging anatomical sites in aesthetic medicine due to its thin dermis, complex vascularity, and susceptibility to oedema and contour irregularities. While hyaluronic acid (HA) fillers remain the gold standard for volumetric correction, their limitations in skin quality enhancement and risk of complications such as Tyndall effect and malar oedema have driven interest in regenerative alternatives. Polynucleotides (PN), particularly polydeoxyribonucleotides (PDRN), have emerged as bioactive agents capable of promoting dermal remodelling, angiogenesis, and anti-inflammatory responses. This review critically evaluates current evidence comparing PN and HA in periorbital rejuvenation, integrating mechanistic insights, clinical outcomes, and safety considerations. While HA remains superior for structural correction, PN demonstrates consistent improvements in dermal quality parameters, including elasticity, hydration, and fine rhytids, with a favourable safety profile. However, heterogeneity in study design, product formulation, and outcome measures limits the ability to draw definitive conclusions. Future research should prioritise standardised protocols, long-term follow-up, and direct comparative trials to establish optimal treatment algorithms.

## 1. Introduction

### Anatomical Complexity of the Periorbital Region

The periorbital region represents one of the most anatomically and functionally complex areas in aesthetic medicine, and consequently, one of the most challenging to treat predictably. Unlike other facial regions, the infraorbital skin is exceptionally thin, approximately 0.5 mm, and is underpinned by a highly vascularised and dynamic musculoskeletal framework. This unique anatomical configuration not only accelerates the visible manifestations of ageing, but also significantly constrains the therapeutic window for safe and effective intervention [1,2].

From a structural perspective, ageing of the periorbital region is not a singular process but rather a multifactorial interplay between dermal atrophy, ligamentous laxity, redistribution of fat compartments, and alterations in microcirculation. The attenuation of the orbicularis retaining ligament, combined with volume loss in the deep medial cheek fat and pseudo-herniation of orbital fat, contributes to the formation of the tear trough deformity. Simultaneously, dermal thinning and reduced collagen and elastin content result in increased translucency, thereby accentuating underlying vasculature and pigmentation [3,4]. These layered changes culminate in a complex clinical presentation characterised not only by contour irregularities but also by compromised skin quality.

Additional anatomical structures play a critical role in periorbital ageing and treatment outcomes. The orbicularis retaining ligament and tear trough ligament contribute to the characteristic lid-cheek junction deformity through progressive attenuation and tethering. Furthermore, age-related changes in superficial and deep fat compartments, particularly the deep medial cheek fat and sub-orbicularis oculi fat (SOOF), contribute substantially to contour irregularities. The lymphatic drainage network of the lower eyelid and midface is equally important, as disruption or compression of these pathways may predispose patients to persistent oedema following injectable treatments.

**Figure 1:**
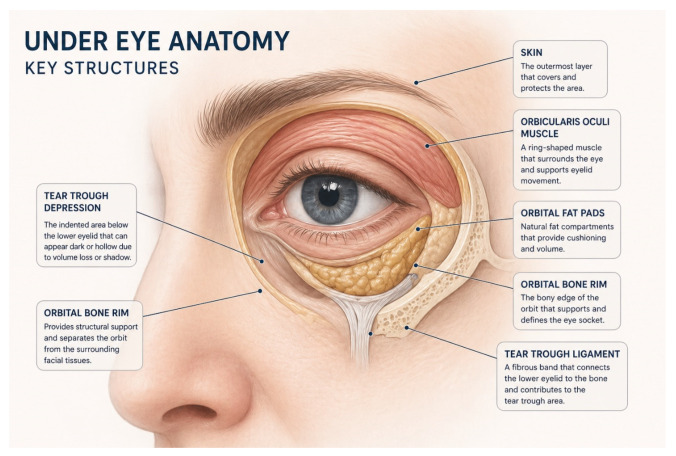
Under-eye (periorbital) anatomical structures, such as orbicularis retaining ligament, tear trough ligament, SOOF, deep medial cheek fat relevant to tear trough deformity and aesthetic treatment.

Figure 1 shows the layered anatomy of the lower eyelid and infraorbital region, including the skin, orbicularis oculi muscle, orbital fat pads, orbital rim, and the tear trough ligament. The tear trough depression is shown as a concavity along the medial infraorbital rim, typically resulting from volume loss, ligamentous tethering, and changes in soft tissue support. Understanding the spatial relationship between these structures is critical for safe and effective delivery of injectable treatments, particularly hyaluronic acid fillers and biostimulatory agents, to restore contour while minimising complications [3]. Critically, this anatomical and physiological fragility renders the periorbital region highly susceptible to treatment-related complications. The dense vascular network and limited lymphatic drainage predispose patients to prolonged oedema, while the thin dermis increases the risk of visible filler placement and the Tyndall effect when using hyaluronic acid. Furthermore, the constant activity of the orbicularis oculi muscle introduces an additional dynamic component, whereby repetitive motion can influence product distribution, longevity, and aesthetic outcomes [4].

This complexity challenges the traditional paradigm of volumetric correction as a primary strategy for periorbital rejuvenation. While hyaluronic acid fillers have demonstrated efficacy in restoring contour deficits, their mechanism of action predominantly based on space occupation and water attraction, does not address the underlying dermal degeneration that contributes to fine rhytids, crepiness, and textural decline. In some cases, volumisation alone may exacerbate aesthetic concerns, particularly in patients with minimal volume loss but significant skin laxity or oedema-prone anatomy [5,6].

**Figure 2:**
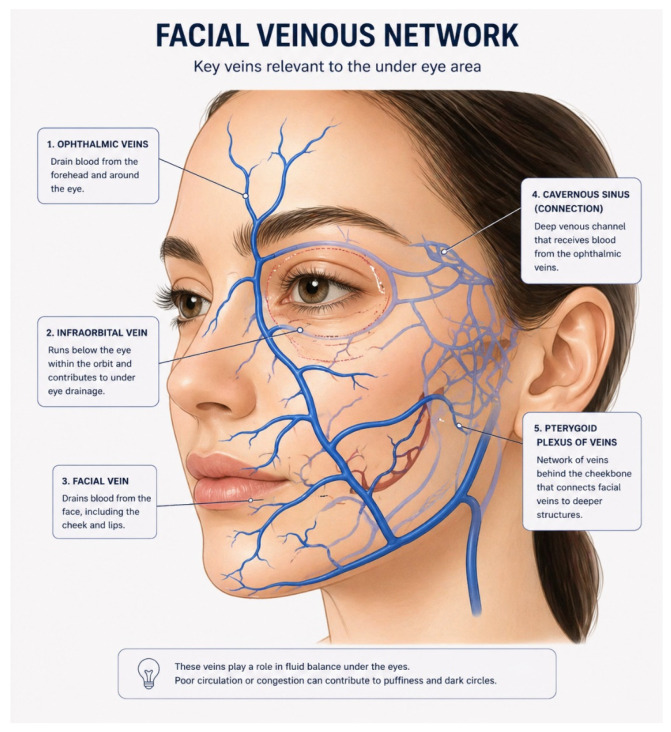
Facial venous anatomy relevant to the periorbital (under-eye) region.

Figure 2 shows the Illustration of the key venous pathways involved in drainage of the midface and infraorbital area, including the ophthalmic veins, infraorbital vein, facial vein, pterygoid venous plexus, and their connection to the cavernous sinus. These interconnected vessels play an important role in periorbital fluid dynamics and are clinically significant in aesthetic practice, as impaired drainage or vascular compromise may contribute to oedema, dark circles, and potential complications during injectable treatments. Beyond the vascular system, lymphatic drainage patterns significantly influence treatment outcomes. Compression of superficial lymphatic channels by filler material has been implicated as a contributing factor in prolonged malar oedema, highlighting the importance of understanding lymphatic anatomy when selecting patients and planning treatment.

Therefore, a critical re-evaluation of treatment approaches in this region is warranted. Contemporary evidence increasingly supports a shift towards regenerative and skin-quality-focused interventions that target the underlying biological processes of ageing rather than solely its structural manifestations. Within this evolving framework, the anatomical complexity of the periorbital region should not be viewed merely as a limitation, but as a determinant guiding more nuanced, multimodal treatment strategies that balance structural correction with dermal regeneration [7,8].

## 2. Methods

### 2.1. Literature Search Strategy

This narrative review was undertaken to evaluate the current evidence regarding the use of hyaluronic acid (HA) fillers and polynucleotides (PN) for periorbital rejuvenation. A structured literature search was conducted using PubMed/MEDLINE, Scopus, Web of Science, and Google Scholar databases to identify relevant publications published between January 2015 and March 2026. The search combined Medical Subject Headings (MeSH) and free-text terms including: “periorbital rejuvenation,” “under-eye rejuvenation,” “tear trough,” “hyaluronic acid filler,” “dermal filler,” “polynucleotide,” “polydeoxyribonucleotide,” “PDRN,” “skin quality,” “regenerative aesthetics,” and “periocular rejuvenation.”.

### 2.2. Study Selection

Articles were screened for relevance based on title and abstract, followed by full-text review where appropriate. Priority was given to clinical trials, randomised controlled studies, systematic reviews, meta-analyses, consensus statements, and observational studies evaluating clinical outcomes, safety profiles, mechanisms of action, treatment protocols, or patient satisfaction associated with HA fillers and PN therapies in the periorbital region. Additional relevant publications were identified through manual screening of reference lists from eligible articles.

### 2.3. Eligibility Criteria

Studies were included if they:

- Evaluated HA fillers, PN, or PDRN-based treatments for periorbital or periocular rejuvenation;
- Reported clinical, aesthetic, mechanistic, or safety outcomes;
- Were published in peer-reviewed journals;
- Were available in the English language.

Studies were excluded if they:

- Focused on non-periorbital anatomical regions without relevant periocular data;
- Were conference abstracts without full publications;
- Were animal-only studies unless providing important mechanistic evidence;
- Lacked sufficient methodological detail or outcome reporting.

### 2.4. Data Synthesis

Given the heterogeneity of study designs, treatment protocols, outcome measures, and PN formulations, a quantitative meta-analysis was not considered appropriate. Findings were therefore synthesised narratively, with emphasis placed on treatment mechanisms, clinical efficacy, safety considerations, patient selection, and emerging treatment algorithms. Particular attention was given to studies directly comparing PN and HA treatments, although the authors acknowledge that comparative evidence remains limited.

### 2.5. Hyaluronic Acid Fillers in Periorbital Rejuvenation

Hyaluronic acid (HA) fillers have long been regarded as the cornerstone of minimally invasive periorbital rejuvenation, primarily due to their capacity to restore volume, their relative safety profile, and the availability of enzymatic reversal with hyaluronidase. Their widespread adoption is underpinned by predictable rheological properties, biocompatibility, and the immediate aesthetic improvement they provide in correcting contour deformities such as the tear trough [9,10]. However, despite these advantages, the application of HA fillers in the periorbital region remains one of the most technically demanding procedures in aesthetic practice, and their limitations become particularly evident when examined through a critical anatomical and functional lens.

The primary mechanism of HA fillers’ space occupation combined with hydrophilic expansion renders them highly effective for structural augmentation. In the context of tear trough deformity, this allows for the restoration of the lid–cheek junction and improvement in shadowing. Nevertheless, this same hydrophilic property, which contributes to their volumising effect, also represents a fundamental drawback in the periorbital region. The propensity of HA to attract water can exacerbate fluid retention in an area already predisposed to lymphatic stasis, thereby increasing the risk of persistent malar oedema [11,12]. This is particularly problematic in patients with pre-existing infraorbital puffiness or compromised lymphatic drainage, where even technically precise injections may result in suboptimal or unpredictable outcomes.

**Figure 3:**
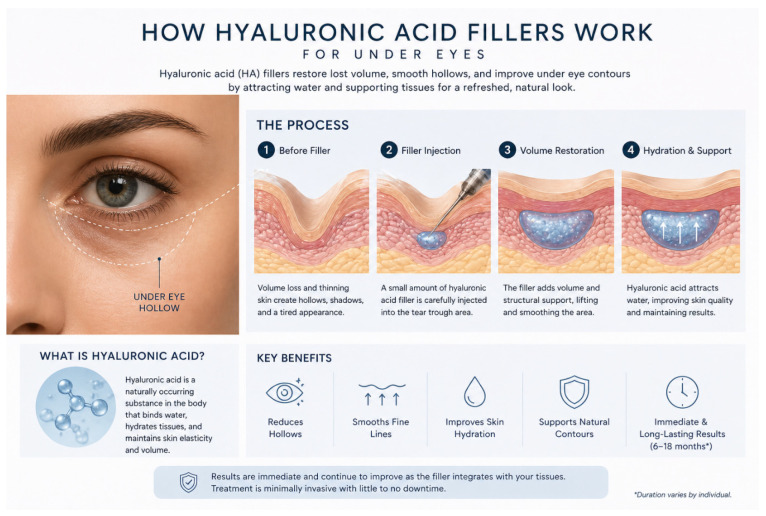
Mechanism of action of hyaluronic acid fillers in periorbital rejuvenation.

The process by which hyaluronic acid (HA) fillers restore under-eye volume and contour. Following injection into the tear trough region, HA provides immediate structural support and volumisation, reducing hollowing and shadowing. In addition, its hydrophilic properties promote water retention, enhancing skin hydration and elasticity. These combined effects result in a rapid, visible improvement in periorbital appearance, with outcomes that may be maintained for several months depending on product characteristics and patient factors as seen in Figure 3.

Furthermore, the thin dermal architecture of the periorbital skin amplifies the visibility of the superficially placed filler. The Tyndall effect, characterised by bluish discolouration due to light scattering, remains a well-documented complication and is largely a consequence of inappropriate depth of placement or unsuitable product selection. While this is often cited as a technical error, it also reflects the narrow margin for error inherent to this anatomical region. Even in experienced hands, achieving the ideal plane consistently can be challenging, particularly given anatomical variability between patients [13,14].

Beyond aesthetic complications, the safety profile of HA fillers in the periorbital region warrants careful consideration. Although rare, vascular occlusion and subsequent visual compromise represent the most serious adverse events associated with filler injections. The proximity of the infraorbital and angular arterial systems, coupled with potential anastomoses with the ophthalmic circulation, necessitates a high level of anatomical knowledge and procedural caution. While the reversibility of HA with hyaluronidase provides a degree of clinical reassurance, it should not be misconstrued as mitigation of risk, but rather as a reactive measure following complication [5,15]. Schematic overview illustrating the primary aesthetic concerns addressed by under-eye fillers, including dark circles, periorbital puffiness, tear trough hollowing, fine lines and creases, and loss of structural support. Hyaluronic acid fillers act by restoring volume, improving contour, and enhancing skin hydration, leading to a smoother and more refreshed appearance.Clinical outcomes are typically immediate, with results lasting several months depending on product characteristics and patient-specific factors.

Critically, HA fillers do not directly address the intrinsic changes associated with periorbital ageing at the dermal level. While they effectively correct volume deficits, they have limited impact on skin quality parameters such as elasticity, hydration balance, and fine textural irregularities. In patients where the primary concern is crepiness or dermal thinning rather than structural hollowing, volumisation alone may fail to achieve satisfactory outcomes and, in some cases, may even accentuate irregularities through overfilling or fluid retention [16].

This disconnect between mechanism and clinical need highlights a broader limitation in the traditional filler-centric approach to periorbital rejuvenation. Increasingly, there is recognition that optimal outcomes in this region require a more nuanced strategy that integrates both structural and regenerative modalities. Within this context, HA fillers retain an essential role in restoring anatomical contour, but their use must be carefully tailored, often in combination with treatments that target dermal quality and tissue health [17,18]. While hyaluronic acid fillers remain indispensable in the management of periorbital volume loss, their application is constrained by anatomical sensitivity, risk of complications, and limited influence on underlying skin biology. Therefore, a critical appraisal of their role underscores the need for judicious patient selection, precise technique, and, importantly, the incorporation of adjunctive therapies that address the multifactorial nature of periorbital ageing.

### 2.6. Polynucleotides in Periorbital Rejuvenation

Polynucleotides (PN), most commonly formulated as polydeoxyribonucleotides (PDRN), have emerged as a biologically driven alternative within aesthetic medicine, reflecting a broader paradigm shift from purely volumetric correction toward regenerative and tissue-modulating therapies [19,20]. Unlike hyaluronic acid fillers, which act predominantly through mechanical augmentation, PN exert their effects at a cellular and molecular level, targeting the fundamental processes underlying dermal ageing. This distinction is particularly relevant in the periorbital region, where structural augmentation alone often fails to address the multifactorial nature of aesthetic decline [21].

**Figure 4:**
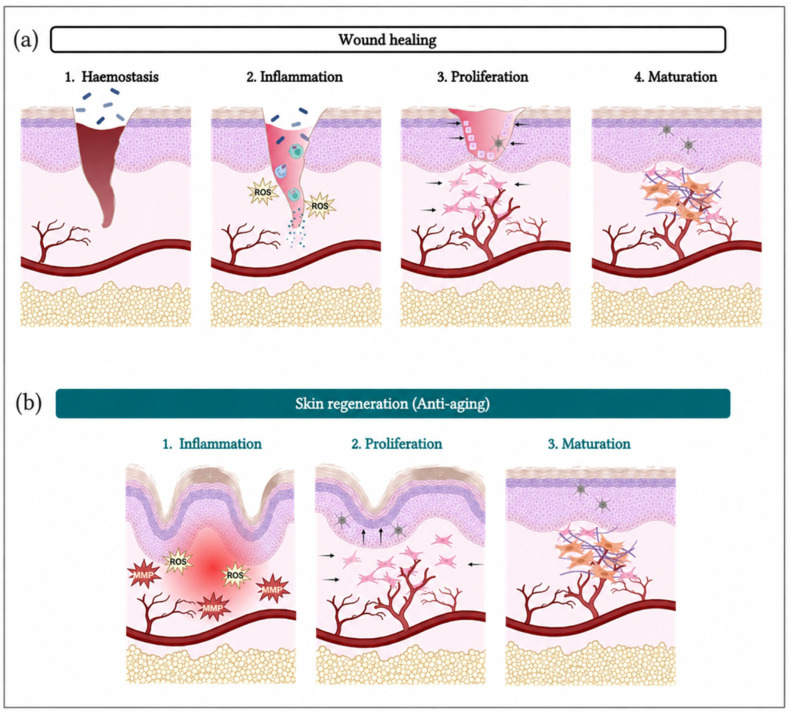
Comparison of classical wound healing and skin regeneration (anti-ageing) processes [3]. Figure 4 shows the schematic representation of the sequential phases of tissue repair. (**a**) Conventional wound healing progresses through four overlapping stages: haemostasis, inflammation, proliferation, and maturation, characterised by clot formation, inflammatory cell infiltration, neovascularisation, and extracellular matrix remodelling. (**b**) Skin regeneration associated with anti-ageing therapies follows a similar but modulated pathway, primarily involving controlled inflammation, enhanced proliferation, and organised maturation, with reduced oxidative stress (ROS) and matrix degradation (MMPs), promoting collagen remodelling, improved vascularisation, and restoration of dermal structure and function [3].

Mechanistically, PN function through activation of the A2A adenosine receptor, initiating a cascade of downstream effects that include fibroblast proliferation, increased synthesis of collagen types I and III, enhanced elastin production, and stimulation of angiogenesis. In parallel, PN engages nucleotide salvage pathways, facilitating cellular repair and regeneration while exerting anti-inflammatory effects [22,23]. These combined actions contribute to improved dermal integrity, hydration, and microcirculatory function parameters that are central to the restoration of skin quality rather than volume. From a theoretical standpoint, this positions PN as a more physiologically aligned intervention in areas characterised by thin, delicate tissue such as the infraorbital region. While these biological effects have been consistently demonstrated in preclinical and laboratory studies, translation into clinically meaningful aesthetic improvements remains incompletely established, and current evidence suggests predominantly modest improvements in skin quality parameters rather than dramatic aesthetic correction as shown in Figure 4.

Clinically, the application of PN in periorbital rejuvenation has been associated with improvements in fine rhytides, crepiness, and overall skin texture, often with a more subtle and progressive aesthetic outcome compared to HA fillers. This gradual onset of effect, while sometimes perceived as a limitation by patients seeking immediate results, may in fact represent a more naturalistic approach to rejuvenation, avoiding the risks of overcorrection and unnatural contour [24]. Importantly, the low volumetric impact of PN reduces the likelihood of complications associated with fluid retention, such as malar oedema, which remains a persistent challenge in HA-based treatments [25]. Importantly, many of the proposed regenerative mechanisms remain mechanistic hypotheses supported primarily by experimental models. Clinical studies have demonstrated improvements in hydration, elasticity, and wrinkle severity; however, direct evidence linking these outcomes to specific molecular pathways remains limited.

**Figure 5:**
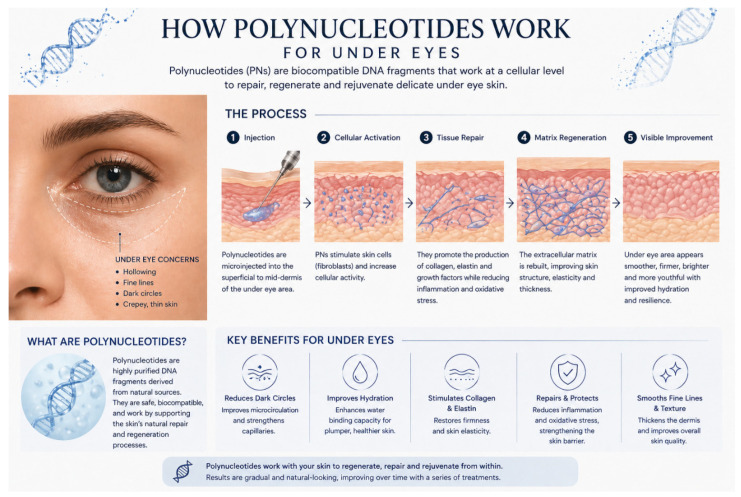
Mechanism of action of polynucleotides in periorbital rejuvenation.

The stepwise mechanism of polynucleotide (PN) therapy in the under-eye region, from intradermal injection to cellular activation, tissue repair, and extracellular matrix regeneration. PN stimulate fibroblast activity, enhance collagen and elastin synthesis, and promote angiogenesis while exerting anti-inflammatory and antioxidant effects. These processes lead to progressive improvements in skin quality, hydration, elasticity, and reduction of fine lines and dark circles, resulting in a smoother and more rejuvenated periorbital appearance over time as shown in Figure 5.

However, despite these promising attributes, a critical evaluation of PN reveals several limitations that temper its current clinical positioning. Firstly, the evidence base, although growing, remains relatively limited in scale and standardisation. Variability in PN formulations ranging in molecular weight, concentration, and purification techniques introduces heterogeneity that complicates direct comparison between studies and limits reproducibility of outcomes [25]. Furthermore, treatment protocols are not yet standardised, with differences in injection depth, frequency, and total treatment sessions contributing to inconsistent clinical results.

The cellular mechanisms and clinical outcomes of polynucleotide (PN) treatment in the under-eye region. PN act by stimulating fibroblast activity, enhancing collagen and elastin synthesis, and promoting dermal regeneration. These processes contribute to improved skin quality, hydration, and elasticity, with subsequent reduction in fine lines, dark circles, and textural irregularities. Clinical effects develop gradually over time, resulting in subtle, natural-looking improvements in periorbital appearance as shown in Figure 5.

Secondly, the magnitude of clinical improvement associated with PN, while statistically significant in many studies [26], is often modest when compared to the immediate and more pronounced effects achieved with HA fillers. PN do not provide structural support and therefore cannot address moderate to severe tear trough deformities or significant volume loss. As such, their role is inherently limited to skin quality enhancement rather than contour correction, necessitating combination approaches in a large proportion of patients [26,27].

Another important consideration is the temporal nature of PN outcomes. The regenerative processes they stimulate require time to manifest, and maintenance treatments are typically necessary to sustain results [28]. This has implications for patient compliance, cost-effectiveness, and overall treatment planning. Additionally, while PN are generally well tolerated, with adverse events largely confined to transient injection-site reactions, long-term safety data particularly in the periorbital region remain sparse. From a critical perspective, PN should not be viewed as a replacement for established modalities such as HA fillers, but rather as a complementary tool within a broader, multimodal treatment strategy. Their greatest value lies in addressing the qualitative aspects of ageing improving skin texture, elasticity, and hydration particularly in patients where volumisation is either unnecessary or carries an elevated risk of complications. In this context, PN align well with contemporary trends in aesthetic medicine that prioritise subtle, biologically harmonious outcomes over overt structural alteration [29].

### 2.7. Polynucleotides in Comparison with Hyaluronic Acid

Lee et al., 2022 [30], evaluates (PN) compared with hyaluronic acid (HA) for periorbital rejuvenation, (PN) fillers have recently emerged as a novel option for periorbital rejuvenation, demonstrating comparable efficacy to (HA) fillers in improving overall aesthetic outcomes. In a randomized, double-blind, split-face trial, PN fillers showed similar improvements in visual analogue and global aesthetic scores when compared with non-crosslinked HA. Notably, PN treatment demonstrated relatively greater improvements in skin elasticity, hydration, surface roughness, and pore volume over time. Both treatments were well tolerated, with no significant adverse events reported, supporting the safety and clinical applicability of PN fillers in the periocular region [30]. While split-face study designs offer the advantage of reducing inter-individual variability and enabling direct intra-patient comparison, they are inherently constrained by challenges related to blinding and potential performance bias, which must be considered when interpreting outcomes.

Importantly, not all periorbital concerns appear equally responsive to PN therapy. Current evidence suggests that PN may be particularly beneficial in patients presenting with dermal thinning, crepey skin, fine rhytides, reduced skin quality, and signs of age-related tissue degeneration. In these patients, the regenerative effects of PN may contribute to improvements in dermal structure and overall skin quality. Conversely, patients whose primary concern is tear trough hollowing, volume loss, or contour deficiency may experience limited aesthetic improvement with PN monotherapy, as PN provide minimal volumetric support. Consequently, appropriate patient selection is critical when incorporating PN into periorbital treatment protocols.

Both polynucleotide (PN) and hyaluronic acid (HA)-based injectables improve periorbital appearance, but studies suggest different patterns: PN/PN–HA tends to drive gradual skin-quality regeneration, while HA fillers give rapid, visible hollow correction [30]. In a randomized split-face trial (27 subjects), PN vs. non-crosslinked HA in the periorbital area showed no significant difference in GAIS or visual analog scores; both were effective and safe. Objective measures favored PN, elasticity, hydration, roughness, and pore volume improvement rates were generally higher or more sustained with PN [24,30]. A clinical PN filler study for periorbital wrinkles found LCL (crow’s feet) improvement ~37–39% at 16–28 weeks, with maintained hydration gains and no major adverse events [31]. A broader review reports PN often yields greater wrinkle-depth reduction and longer duration than HA in periocular and crow’s feet studies, while both improve elasticity and hydration. A broader review reports PN often yields greater wrinkle-depth reduction and longer duration than HA in periocular and crow’s feet studies, while both improve elasticity and hydration [19]. It should be emphasised that direct comparative evidence between PN and HA remains limited, with most available data derived from a small number of comparative studies and indirect comparisons across heterogeneous populations and treatment protocols.

Large HA infraorbital trials report GAIS responder rates ~87–98% from 3 to 12 months, with patients and investigators aligning closely [26,32]. Real-world data with a periorbital HA filler showed >70% “improved/much improved” GAIS at 3 months, >90% by investigators, with effects visible up to 12 months [24]. Non-crosslinked HA tear-trough treatment achieved GAIS responder rates ~86–90% at 6 months, with high satisfaction [33].

Evidence indicates that both PN and HA significantly improve periorbital appearance. HA fillers reliably produce rapid, pronounced hollow correction with high early GAIS response rates (often 80–90%+ within months) and durability up to 6–18 months in many studies as shown in Table 1. PN-based treatments, while similarly effective on GAIS in head-to-head periocular trials, tend to show a more gradual, regenerative trajectory, with particular strengths in skin elasticity, texture, and potentially longer-lasting wrinkle improvements.

### 2.8. Safety of PN vs. HA in the Periorbital Area

Evidence consistently shows that polynucleotides (PN) and hyaluronic acid (HA) differ more in safety profile than in efficacy, especially around the eyes. Across a systematic review of 219 PN-treated patients, side effects were generally mild and transient, with no serious complications reported [25]. Reported reactions are mainly local injection-site events (erythema, oedema, bruising/burning) that resolve spontaneously; expert consensus notes erythema, burning within 12 h, and small hematomas as the most common issues [5,25]. Real-world PN HPT data (66 patients, 106 treatment areas) and large PN-HPT + HA series (218 questionnaires, 654 infiltrations) both report no serious or unexpected adverse events [19,37]. Periorbital PN case series showed only mild swelling, discomfort, minor bruising at 2 days, and no delayed/persistent events such as nodules, prolonged oedema, Tyndall-like discoloration, or infection up to 6 months [38].

Reviews of periocular HA stress that most complications are also immediate, mild injection-related reactions (erythema, oedema, bruising/hematoma) [39,40]. However, persistent or late malar/lower-eyelid oedema, blue discoloration (Tyndall effect), contour irregularities, filler migration, and chronic oedema are well documented and may appear weeks to years later [39,40,41,42]. Long-term follow-up of 147 patients found malar oedema in 11%, blue-grey dyschromia (Tyndall-like) in 31.3%, and contour irregularities in 30.5%; 90% were mild and often required no intervention [41]. Duplex ultrasound case series and late-onset oedema reviews link malar oedema to veno-lymphatic compression by filler and often require hyaluronidase and sometimes surgery for resolution [41,42,43]. Periorbital vascular events (retinal/ophthalmic artery occlusion) are rare but vision-threatening, with multiple reports of visual loss after facial HA injections; outcomes are often poor despite treatment, reflecting the anatomical vulnerability and need for meticulous technique and emergency hyaluronidase access [44,45].

Current evidence supports PN as having a very favourable, mainly procedure-related and self-limiting safety profile, with no serious PN-attributed events reported so far. HA fillers in the periorbital region are overall safe and effective but carry distinct, sometimes persistent complications (notably malar oedema and Tyndall effect) and a rare yet severe risk of vascular occlusion and visual loss, driven by regional anatomy rather than product alone.

### 2.9. Mechanistic Superiority Versus Clinical Reality

Evidence supports PN as a biologically active, regenerative agent, but clinical facial outcomes are generally moderate and slow-onset, and not directly comparable to the rapid volumizing effects of HA fillers. PN and PN–HA formulations stimulate fibroblast proliferation, collagen production, ECM remodelling, angiogenesis, and migration in multiple in-vitro and animal models [24,39,46]. PN and PN-HA also show anti-inflammatory and antioxidant effects and support soft-tissue healing and microcirculation [47,48]. Filler and wound-healing studies confirm enhanced dermal regeneration and collagen deposition versus HA alone in preclinical systems [24,39,49]. In periorbital split-face trials, PN and non-crosslinked HA achieved similar GAIS/VAS improvements, with PN showing somewhat better elasticity, hydration, roughness and pore metrics, but overall changes remained in the “improved” rather than transformative range [50,51]. A PN periorbital trial reported LCL wrinkle improvement of ~37–39% at 16–28 weeks, with modest hydration gains and no major adverse events [51]. A systematic review (9 studies, 219 patients) describes statistically significant but moderate improvements in wrinkles, texture, and elasticity, with mostly mild side effects and “moderate to high” satisfaction [25]. Across current studies, PN shows clear regenerative biology but only moderate, gradual aesthetic gains, especially versus the immediate visual impact of HA fillers. Evidence supports a complementary strategy: HA for structural correction and rapid satisfaction; PN primarily to improve skin quality and to prime or refine outcomes, rather than to replace HA in volume-loss indications. Although PN demonstrates compelling biological activity through stimulation of fibroblasts, collagen synthesis, extracellular matrix remodelling, and angiogenesis, caution is required when extrapolating these findings into clinical superiority. Current comparative clinical studies generally report similar global aesthetic outcomes between PN and HA treatments, with differences primarily observed in objective skin-quality parameters rather than overall aesthetic improvement. Consequently, mechanistic superiority should not be assumed to equate to superior clinical effectiveness.

**Figure 6:**
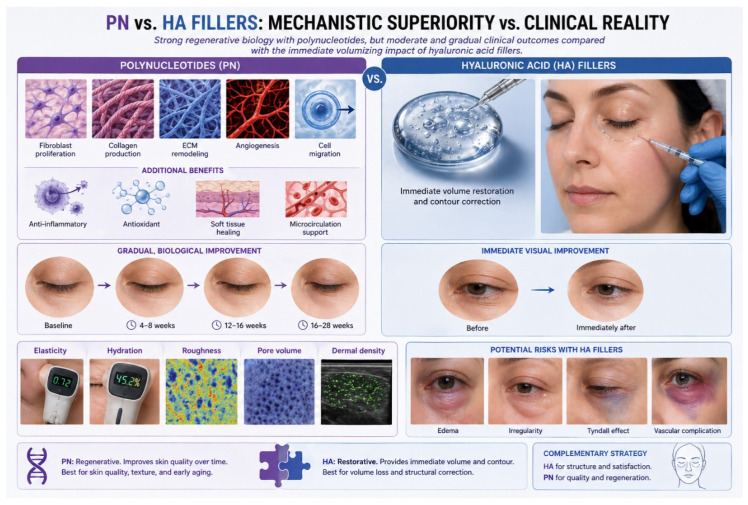
Mechanistic superiority versus clinical reality: polynucleotides versus hyaluronic acid fillers in periorbital rejuvenation.

Figure 6 shows the biological mechanisms and clinical outcomes of polynucleotides (PN) and hyaluronic acid (HA) fillers. PN demonstrate strong regenerative activity, including fibroblast stimulation, collagen synthesis, extracellular matrix remodelling, and angiogenesis, leading to gradual improvements in skin quality over weeks. In contrast, HA fillers provide immediate volumetric correction and contour restoration. Despite robust biological effects, PN yield moderate, progressive clinical changes, whereas HA delivers rapid, visible results but with potential risks such as oedema, irregularities, and Tyndall effect. The figure highlights their complementary roles within an integrated treatment approach.

### 2.10. Clinical Integration Framework

#### Treatment Strategy and Algorithm

Evidence supports using polynucleotides (PN) and hyaluronic acid (HA) as complementary, not competing, tools, with choice and sequencing tailored to the dominant clinical problem. HA remains the workhorse. Reviews and algorithms on infraorbital treatment emphasize HA fillers for groove/hollow correction and eyelid–cheek transition smoothing, with high satisfaction and low serious-complication rates [24,30,52]. PN is repeatedly used as a skin booster for fine infraorbital/periorbital lines and texture in expert consensus and practice surveys [19,53]. PN studies show improvements in wrinkles, elasticity, and texture in periocular and facial sites with good tolerance [42,54]. 

In nasolabial folds, PN-HPT priming followed by cross-linked HA improved wrinkles/texture and prolonged HA volume effect vs. HA alone (https://consensus.app/papers/clinical-efficacy-and-safety-of-polynucleotides-highly-araco-araco/52773887fd665293a934cc54f7830313/, accessed on 26 May 2026). A consensus on the PN-HPT priming paradigm recommends 2–4 PN sessions before fillers to recondition dermis and stabilize filler outcomes [55,56]. A PN-HA regenerative complex showed superior in vitro collagen stimulation vs. other HA boosters and promising periocular case results, supporting multifactorial skin-quality enhancement rather than pure filling [48]. PN consensus guidance suggests face/periocular protocols of 3–4 sessions 2–3-weekly, followed by spaced maintenance; however, optimal long-term intervals remain undefined [42].

### 2.11. Patient Selection

PN is favoured in expert consensus and observational work for delicate, oedema-prone periocular skin and for patients seeking subtle, natural improvements without added volume [42,57]. HA tear trough guidelines stress strict selection (skin quality, vector, oedema tendency), minimal volumes (≤0.5 mL/eye), midface support first, and readiness to reverse with hyaluronidase to avoid irregularities, oedema, and delayed complications [24,56]. PN and PN-HA are associated with gradual, progressive improvements in texture, fine lines, and luminosity over weeks to months, often with minimal downtime and high satisfaction [42]. HA provides immediate post-procedure contour change and rapid GAIS improvement in infraorbital and midface rejuvenation (https://consensus.app/papers/comparison-of-the-effects-of-polynucleotide-and-kim-lee/4170d0a11eb25f5eb702bedf1103d933/, https://consensus.app/papers/optimizing-infraorbital-hollows-treatment-with-bhojani-lynch-berros/f406f94f569f5cf58a93acec0a8b728c/, https://consensus.app/papers/a-prospective-study-on-safety-complications-and-diwan-trikha/1b20d4bb708450b0ade235879602d5bc/, https://consensus.app/papers/hyaluronic-acid-injection-strategy-of-infraorbital-and-zhang-xu/95cf17a4ba525546a79f42868a47a373/, accessed on 30 May 2026), but carries higher risk of visible overfilling or oedema if selection/technique are suboptimal [19,24,56].

Current evidence aligns with a stepwise, patient-specific algorithm: HA fillers for clear volumetric/structural deficits, PN (alone or as PN-HA) for dermal quality and in oedema-prone or subtlety-seeking patients; and sequential PN + HA when both structure and tissue quality require treatment. Such multimodal plans better match the dual structural and biological nature of periorbital ageing while optimizing safety.

**Figure 7:**
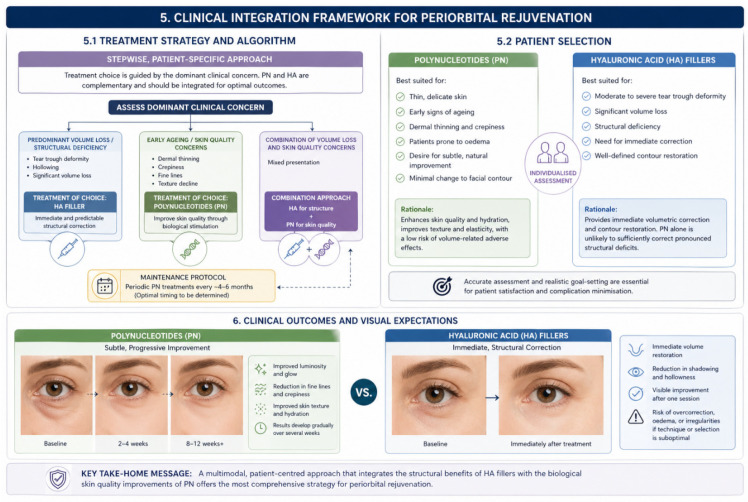
Clinical integration framework for periorbital rejuvenation using polynucleotides and hyaluronic acid fillers.

Figure 7 shows the schematic representation of a patient-centred, stepwise treatment strategy for the management of periorbital ageing. Treatment selection is guided by the dominant clinical concern: hyaluronic acid (HA) fillers are preferred for patients with significant volume loss and structural deficiency, providing immediate contour correction, whereas polynucleotides (PN) are indicated for early ageing changes characterised by dermal thinning, crepiness, and reduced skin quality. In mixed presentations, a combination approach is recommended, with HA used for structural support followed by PN to enhance dermal regeneration and refine outcomes. Appropriate patient selection is essential, with PN favoured in patients with thin skin or oedema-prone tissues, and HA in those requiring more substantial volumisation. The figure also highlights differing clinical outcomes, with PN producing gradual, progressive improvements in skin quality over weeks, and HA delivering immediate volumetric correction, albeit with a higher risk of overcorrection if used inappropriately.

Across current data, PN treatments act as gradual skin-quality enhancers, with subtle, progressive improvements in texture, fine lines and luminosity over weeks to months. HA fillers, by contrast, provide immediate volumetric correction and contour restoration in a single session, with a higher risk of visible overcorrection or irregularity if poorly executed. The literature supports viewing PN and HA as complementary modalities, PN for regenerative refinement, and HA for rapid structural change.

From a clinical perspective, three broad patient categories can be identified. The first includes patients with significant tear trough deformity, volume loss, and structural deficiencies, for whom HA fillers remain the preferred treatment modality. The second includes patients with thin, crepey, photodamaged, or oedema-prone skin, where skin quality deterioration rather than volume loss predominates; these patients may derive greater benefit from PN-based treatments. The third and perhaps most common category comprises patients presenting with both structural and dermal ageing changes. In such cases, combination therapy may offer synergistic benefits, with HA fillers restoring volume and contour while PN improves tissue quality, hydration, elasticity, and dermal regeneration. This integrated approach may provide more comprehensive and natural-looking rejuvenation than either modality alone.

## 3. Limitations of Current Evidence

Interpretation of the current literature should be undertaken cautiously. Many PN studies involve small sample sizes, single-centre designs, and relatively short follow-up periods. Considerable heterogeneity exists between PN formulations with respect to molecular weight, concentration, purification methods, and treatment protocols, limiting direct comparison between studies. Outcome measures are also inconsistent, ranging from subjective aesthetic assessments to objective instrumental measurements. Furthermore, direct head-to-head comparative studies between PN and HA remain scarce, and long-term comparative data extending beyond 12 months are largely unavailable. These limitations reduce the strength of current conclusions and highlight the need for standardised, adequately powered randomised controlled trials.

## 4. Future Research Directions

Current studies on polynucleotides (PN) and hyaluronic acid (HA) in periorbital and facial rejuvenation are promising but are small, heterogeneous, and often exploratory. Several concrete gaps and next steps can be identified. Most PN studies include small samples (often ≤30 patients) with no or limited controls and exploratory statistics. Follow-up in PN facial studies is typically ≤6 months; durability beyond 6–12 months and optimal maintenance intervals are unclear. Standardisation of PN formulations and treatment protocols is essential to enable meaningful comparison across studies and to establish evidence-based clinical guidelines. Long-term follow-up studies extending beyond 12 months are particularly important in assessing the durability of PN-induced dermal changes and determining optimal maintenance intervals. Direct comparative studies between PN and low G′ hyaluronic acid fillers would provide valuable insights into their relative efficacy in the periorbital region. However, the current evidence base remains limited by small sample sizes, methodological heterogeneity, and a lack of robust long-term comparative studies. Additionally, the integration of advanced imaging techniques and ultrasound-guided injections may enhance both safety and precision, representing a promising direction for future clinical practice.

From a practical clinical perspective, current evidence supports a personalised treatment approach rather than a direct substitution of one modality for another. Hyaluronic acid fillers remain the treatment of choice for patients with clinically significant tear trough deformity, volume loss, and structural deficiencies requiring immediate correction. In contrast, polynucleotides appear most beneficial in patients presenting with dermal thinning, crepiness, early ageing changes, or a predisposition to oedema, where improvements in skin quality rather than volumisation are desired. For many patients, the available literature suggests that optimal outcomes may be achieved through a combined approach, utilising HA for structural support and PN for dermal regeneration and tissue quality enhancement. However, these recommendations should be interpreted cautiously given the limited availability of direct comparative studies.

## 5. Conclusions

Polynucleotides represent a biologically driven advancement in periorbital rejuvenation, targeting key pathways involved in dermal ageing, including collagen synthesis, extracellular matrix remodelling, and tissue repair. Despite these mechanistic advantages, current clinical evidence indicates that their aesthetic effects are modest, gradual in onset, and not directly comparable to the immediate volumetric correction achieved with hyaluronic acid (HA) fillers. Rather than serving as a replacement, PN should be positioned as a complementary modality within a multimodal treatment framework. HA fillers remain the cornerstone for correcting volume loss and structural deficiencies, while PN contribute to improvements in skin quality, particularly in thin, delicate, or oedema-prone periorbital tissues. A critical synthesis of the available literature supports an integrated, patient-specific approach, in which structural augmentation and biological regeneration are strategically combined to optimise outcomes. Clinically, PN appear most suitable for patients exhibiting dermal ageing and compromised skin quality, whereas HA fillers remain preferable for structural correction. For patients demonstrating both tissue degeneration and volume loss, current evidence supports a combined treatment strategy to address the multifactorial nature of periorbital ageing. Future advancements in this field will likely be driven by refined combination protocols and higher-quality, long-term clinical data. Until such evidence is established, clinical decision-making should remain grounded in a balanced, evidence-based understanding of both the capabilities and limitations of regenerative therapies.

## Figures and Tables

**Table 1:** GAIS and Response Trajectory (Illustrative HA vs. PN).

Treatment Type	Typical GAIS/Aesthetic Response Pattern	Citations
**Non-crosslinked/crosslinked HA fillers**	High early GAIS responder rates (often >80–90%) from 1–3 months, sustained in many cases to 6–12+ months	[32,34,35,36]
**PN monotherapy**	Demonstrated wrinkle and hydration improvement over 4–6+ months; GAIS similar to HA in direct periocular comparison	[19,31]
**PN–HA complexes**	Case-level and mechanistic data suggest fast visible change plus regenerative skin-quality gains	[24,26]

## Data Availability

No new data were created or analysed in this study. Data sharing is not applicable to this article.
